# Analysis of a series of Italian APECED patients with autoimmune hepatitis and gastro-enteropathies

**DOI:** 10.3389/fimmu.2023.1172369

**Published:** 2023-06-30

**Authors:** Giorgia Paldino, Maria Felicia Faienza, Marco Cappa, Andrea Pietrobattista, Donatella Capalbo, Mariella Valenzise, Vito Lampasona, Annamaria Cudini, Elena Carbone, Olivia Pagliarosi, Giuseppe Maggiore, Mariacarolina Salerno, Corrado Betterle, Alessandra Fierabracci

**Affiliations:** ^1^ Bambino Gesù Children’s Hospital, IRCCS, Rome, Italy; ^2^ Department of Precision and Regenerative Medicine and Ionian Area, University of Bari “Aldo Moro”, Bari, Italy; ^3^ Research Unit for Innovative Therapies in Endocrinopathies, Bambino Gesù Children’s Hospital, IRCCS, Rome, Italy; ^4^ Hepatology, Gastroenterology and Nutrition Unit, Bambino Gesù Children’s Hospital, IRCCS, Rome, Italy; ^5^ Pediatric Endocrinology Unit, Department of Mother and Child, University Hospital Federico II, Naples, Italy; ^6^ Department of Human Pathology of Adulthood and Childhood, Messina University, Messina, Italy; ^7^ San Raffaele Hospital, IRCCS, Milan, Italy; ^8^ Pediatric Endocrinology Unit, Department of Translational Medical Sciences, University of Naples Federico II, Naples, Italy; ^9^ Clinical Immunology, Padua University, Padua, Italy

**Keywords:** APECED, autoimmune hepatitis, autoimmune gastro-enteropathy, AIRE, diagnostic criteria, autoimmune gastroenteropathy

## Abstract

**Introduction:**

Autoimmune polyendocrinopathy-candidiasis-ectodermal dystrophy (APECED) syndrome is a rare monogenic disease determined by biallelic mutations in *AIRE* gene, which encodes a transcription factor essential for central immune tolerance. Classic diagnosis is determined by the presence of two of the main APECED clinical diseases: chronic mucocutaneous candidiasis, chronic hypoparathyroidism, and Addison’s disease. Non-endocrine autoimmunity, involving the liver, intestine, eyes, and kidneys, is generally reported in a minority of European patients, while American APECED patients have a higher tendency of developing organ-specific non-endocrine manifestations early in life. This observation led to the revision of the diagnostic criteria to permit earlier diagnosis based on the appearance of one classic triad symptom or one non-classical manifestation at a young age in the presence of IFNωAbs or *AIRE* mutations (Ferre-Lionakis criteria).

**Patients and methods:**

We analyzed the clinical, genetic, and autoantibody (Ab) profiles in a series of 14 pediatric Italian APECED patients with gastrointestinal manifestations (seven male and seven female patients). Ten patients presented hepatitis (APECED-associated hepatitis (APAH)), while seven were affected by constipation, diarrhea, and malabsorption. Four patients had developed APAH before classic triad symptoms.

**Results:**

Based on the age of appearance of non-endocrine manifestations including APAH and gastro-enteropathy, the Ferre-Lionakis criteria would have allowed an expedited diagnosis in 11/14 patients. Abs to tryptophan hydroxylase (TPHAb) and hepatic aromatic l-amino acid decarboxylase (AADC) were significantly associated with APECED patients of the present series. Abs to cP4501A2 were detectable in the serum of 4/8 patients with APAH, and Abs to cP4502A6 were detectable in 3/8 patients. AADC Abs tested positive in 5/7 patients, which is indicative of gastrointestinal dysfunction in APECED and TPHAb in 5/7 patients with gastrointestinal dysfunction. IFNAb was significantly associated with the syndrome.

**Conclusion:**

Although Ferre-Lionakis expanded criteria applied to the American cohorts of APECED patients would require validation in independent large cohorts of European patients, the results of this study emphasize the importance to evaluate the presence and the age of appearance of APAH and autoimmune enteropathy even in European cohorts for an earlier APECED diagnosis. An earlier APECED diagnosis would also allow the prevention of episodes of life-threatening hypocalcemic seizures and adrenal crisis, which are the main manifestations of undiagnosed APECED.

## 1 Introduction

Autoimmune-polyendocrinopathy-candidiasis-ectodermal dystrophy (APECED, OMIM240300) syndrome is a rare monogenic disease due to biallelic mutations in autoimmune regulator (*AIRE*) gene (1). Classic diagnostic criteria are the presence of two of the three main APECED symptoms: chronic mucocutaneous candidiasis (CMC), chronic hypoparathyroidism (CH), and Addison’s disease (AD). Non-endocrine autoimmunity involving the liver, stomach, intestine, eyes, and kidneys is generally reported in a minority of patients (1).

Although it depends on the series analyzed, approximately 25% of APECED patients in different populations are affected by gastro-enteropathy (GE) manifestations (2) including celiac disease, cystic fibrosis, pancreatic exocrine insufficiency (PEI), autoimmune intestinal disease (2) and loss of enterochromaffin cells (3, 4), intestinal infections, gastritis due to an autoimmune attack against gastric parietal cells leading to gastric atrophy, and pernicious anemia (5). Circulating autoantibody (Abs) against parietal cells (anti-gastric parietal cells (PCAs)), i.e., anti-sodium potassium channel molecule and anti-intrinsic factor (IFA), are detectable. Candida esophagitis is characterized by pseudomembranotic lesions, erosion, and ulceration with retrosternal pain while swallowing and dysphagia (6, 7). Intestinal candidiasis may also be responsible for watery diarrhea and malabsorption (7). Autoimmune enteropathy is characterized by small intestinal villous atrophy, protracted diarrhea, no response to exclusion diets, and no severe immunodeficiency (2, 3, 8). Abs to tryptophan hydroxylase (TPHAb), an intestinal autoantigen expressed in serotonin-producing cells in the central nervous system and the intestine, were detected in APECED (8). Lymphangiectasia of the small intestine may also be responsible for steatorrhea in APECED patients (9).

Among the other non-endocrine manifestations, APECED-associated hepatitis (APAH) has been described to occur at different prevalence in large series of APECED patients among different ethnicities (reviewed (rev) in 10). Further, APAH is the first manifestation in less than 2% of APECED patients (rev in 11). Abs are detected against hepatic aromatic l-amino acid decarboxylase (AADC), cytochrome P450 family 1 subfamily A member 2 (CYP1A2), histidine decarboxylase (HDC), bactericidal permeability-increasing fold-containing B1, TPH, and p450 21-hydroxylase (21-OH, CYP21A2). No liver-specific autoantigen has been so far identified in all patients with APAH, and it has not been possible to specifically distinguish APAH from classical autoimmune hepatitis (AIH) (10). The homozygous c.967_979del13 *AIRE* mutation is the most frequently associated genotype to APAH development (10).

American APECED patients showed enrichment of organ-specific non-endocrine manifestations early in life when compared to European cohorts (11). Between 40% and 80% of the patients had non-major disorders at onset, while the occurrence of classical manifestations was delayed (11). This observation led to the revision of the diagnostic clinical criteria (11), which would permit earlier diagnosis based on the appearance of one classic triad (CMC, AD, and CH) (occurring below 20 years of age) and one non-classical manifestation (enamel hypoplasia, periodic fever with rash, non-infectious keratitis, or autoimmune hepatitis) at a young age in presence of IFNωAb or *AIRE* mutations (11, 12). In light of the foregoing, in this manuscript, we report and discuss the clinical, genetic, and Ab profile in a series of 14 pediatric Italian APECED patients with APAH or gastrointestinal manifestations highlighting common features and evaluating whether their early enrichment would allow an expedited clinical diagnosis of APECED similar to the American cohort.

## 2 Patients and methods

### 2.1 Study population

The overall population consists of 14 cases (seven male and seven female patients): 10 unrelated patients (**Table 1**, Nos. 1, 2, and 7–14) and two couples of siblings (Nos. 3–4 and 5–6). All patients were recruited from continental Italy except for patient No. 8, who was recruited from Sicily. Although all the patients were referred and diagnosed in childhood, eight patients (Nos. 4–8 and 11–13) are currently adults.

**Table 1:** Clinical, genetic, and immunological characteristics of the 14 APECED patients.

Pt	Gender	Age (years)	Age at referral (years)	Major APECED diseases	Other clinical manifestations	Auto Abs	IFN Abs	*AIRE* gene pattern*/protein	Therapy
1. (13)	M	6.5	5.4		Acute liver failure (5), hypergammaglobulinemia, autoimmune hepatitis (5), onychodystrophy (6)	**AADC, IA2Ab, SMA, ACA, SCC Ab, 21OHAb, AADC**, **cP4501A2Ab pos** TgAb, TPOAb, IAA, GADAb, ZnT8Ab, AGA, ENA, ANA, ANCA, AMA, ARA, dsDNA, LKMAb, LC1Ab, RAb, SLA-IgG, PCA, 17αOHAb, HarmoninAb, VillinAb, TPHAb, cP4502D6Ab, cP4502A6Ab neg	**IFNω IFNα4 pos**	het c.967_979del13 (L323SfsX51) het c.1259_1260delTG (V420GfsX3)	Prednisone Omeprazole Azathioprine Ursodeoxycholic acid Calcifediol
2.	F	14.1	7	CH (7), CMC (13)	HT (7), vernal keratoconjunctivitis (10), vitiligo (11), selective partial IgA deficit (12), autoimmune hepatitis (13), hypertransaminasemia, hypergammaglobulinemia	**TPHAb, AADC, TgAb, TPOAb, ANA, ACA, 17αOHAb, SSA, cP450c21Ab pos** TRAb, IAA, GADAb, IA2Ab, TRGAb, ANCA, SMA, ARA, dsDNA, LKMAb, LC1Ab, RAb, SLA-IgG, PCA, IFA, SCCAb, HarmoninAb, VillinAb, cP4501A2Ab, cP4502D6Ab, cP4502A6Ab neg	**IFNω IFNα4 pos**	het c.769C>T (R257X) het c.1616C>T (P539L)	Prednisone Hydrocortisone Fludrocortisone Ursodeoxycholic acid Azathioprine Calcitriol Levothyroxine (LT4) Lansoprazole Fluconazole
3. (14)	M	13.2	9.8		Autoimmune hepatitis (9), hypertransaminasemia, hypergammaglobulinemia	**AADC, ANA, ACA, SMA, IFA, SCCAb**, **cP450c21Ab, AADC, cP4501A2Ab, cP4502D6Ab**, **cP4502A6Ab pos** TgAb, TPOAb, TPH, IAA, GADAb, TRGAb, ENA, ANCA, AMA, ARA, dsDNA, LKMAb, LC1Ab, ASCA, RAb, SLA-IgG, PCA, 17αOH Ab, HarmoninAb, VillinAb, TPHAb neg	**IFNω** **IFNα4**	het c.415C>T (R139X) het c.967_979del13 (L323SfsX51)	Prednisone Lansoprazole Azathioprine
4. (14)	M	20.1	11.6	CH (7), AD (17)	Septic arthritis (3), transient GH deficiency (9), autoimmune hepatitis (12), hypergammaglobulinemia, alopecia (16)	**ACA, LKMAb, PCA, IFA**, **17αOHAb**, **SCCAb**, **cP450c21Ab pos** AADC, TgAb, TPOAb, TPH, IAA, TRGAb, AGA, ANA, ANCA, SMA, AMA, ARA, dsDNA, ASCA, RAb, LC1Ab, SLA-IgG, HarmoninAb, VillinAb, AADC, TPHAb, cP4501A2Ab, cP4502D6Ab, cP4502A6Ab neg	**IFNω**	het c.415C>T (R139X) het c.967_979del13 (L323SfsX51)	Hydrocortisone Calcitriol Fludrocortisone Calcium carbonate Folic acid Acetylsalicylic acid
5. (14, 20)	M	20.9	1.6	CH (5), AD (5), CMC (5.5)	Autoimmune hepatitis (1.5), vitiligo (5), alopecia (5), enamel dysplasia (5), nail pitting (5.5), chronic diarrhea/malabsorption (5.5), alternation constipation/diarrhea, autoimmune gastritis (6), keratoconjunctivitis (6), previous PRES (6), periodic rash-associated fever (7), cholelithiasis (11), GH deficiency (11), primary testicular failure (14), early hypertension (14), kidney cysts (14), nasal polyps (15), keratitis, HT, urticaria	**TPHAb, AADC, Thyroid Ab, PCA, IFA, 17αOHAb, cP4501A2Ab, cP4502A6Ab pos** TRGAb, ICA, GADAb, IA2Ab, ENA, ANA, ANCA, SMA dsDNA, LKM, SCCAb, HarmoninAb, VillinAb, cP4502D6Ab neg	**IFNω IFNα4**	homo IVS1 + 1G>C; IVS1 + 5delG	Calcitriol Calcium LT4 Hydrocortisone Prednisone Fludrocortisone anti-hypertensive therapy Anti-fungal therapy
6. (14, 21)	F	19.4	4	CMC (4), CH (4), AD (15)	Nail pitting (4), alopecia (11), HT, onychodystrophy, alternation constipation/diarrhea	**AADC, Thyroid Ab**, **GADAb**, **IA2Ab, 17αOHAb, 21OHAb pos** TRGAb, ICA, ENA, dsDNA, PCA, IFA, SCCAb, HarmoninAb, VillinAb, TPHAb, neg	**IFNω IFNα4 pos**	homo IVS1 + 1G>C; IVS1 + 5delG	Calcitriol Calcium LT4 Hydrocortisone Prednisone Fludrocortisone iron sulfate vitamin C, B6, B12, folic acid supplement Anti-fungal therapy
7. (14)	F	21.3	2	CH (2), CMC (3), AD (3)	Dental enamel dysplasia (3), nail pitting (3), GH deficiency (11.5), primary ovarian failure (12), keratoconjunctivitis (12), chronic constipation (17), frequent abdominal pain, previous *Helicobacter pylori* infection (17), relapsing urticaria, onychodystrophy, hypolacrimia, peripheral retinal degeneration, recurrent headaches	**TPHAb, GADAb**, **SCCAb**, **cP450c21Ab pos** TRGAb, Thyroid Ab, ICA, IA2Ab, ENA, ANA, ANCA, SMA, dsDNA, LKMAb, PCA, IFA, 17αOHAb, HarmoninAb, VillinAb, AADC, cP4501A2Ab, cP4502D6Ab, cP4502A6Ab neg	**IFNω IFNα4 pos**	het c.62C>T (A21V) het c.967_979del13 (L323SfsX51)	Hydrocortisone Fludrocortisone Calcitriol Calcium estrogen and progestin Fecal softeners anti-fungal therapy
8. (15)	M	22.5	1	CMC (1), CH (11)	Autoimmune enteropathy with malabsorption (DQ2/DQ8) (6), hypertransaminasemia (7), iron-deficiency anemia (9), IgA deficit (9), carpal spasms (11)	**TPHAb, AADC, Ro2Ab, pos** Thyroid Ab, 17αOHAb, SCCAb, HarmoninAb, VillinAb, cP4501A2Ab, cP4502D6Ab, cP4502A6Ab neg	**IFNω IFNα4**	homo c.1566 + 2-1566 + 3insT	Calcium carbonate Calcitriol
9.	F	14.2	1	Oral CMC (4), CH (7)	Alopecia (1), onychodystrophy (1), abscesses (4), joint pain (8), chronic mucous diarrhea (9), growth deficit	**TgAb, TPOAb, IAA, ACA, ANA, 21OHAb (cP450c21Ab)**, **cP4502A6Ab pos** GADAb, PCA, IA2Ab, ovaryAb, 17αOHAb, SCCAb, HarmoninAb, VillinAb, AADC, TRGAb, TPH Ab, cP4501A2Ab, cP4502D6Ab neg	IFNω neg **IFNα4 pos**	homo c.64_69delGTGGAC (V22_ D23del)	Calcium carbonate Calcitriol 0.5 rhGH treatment HRT
10.	F	9.5	1	CMC (1), CH (8)	Still’s disease (1), chronic mucous diarrhea (6), psoriatic onychopathy (6), macrocytosis (7), homo MTHFR mutation c.677C>T (7), autoimmune gastritis (9)	**TPHAb, AADC, GADAb**, **ANA**, **IFA pos** TgAb, TPOAb, TRAb, IAA, IA2Ab, ZnT8Ab, ACA, ENA, ANCA, P-ANCA, C-ANCA, SMA, AMA, dsDNA, ASCA, LKMAb, LC1Ab, PCA, 17αOH Ab, SCCAb, HarmoninAb, VillinAb, TRGAb, cP450c21Ab, cP4501A2Ab, cP4502D6Ab, cP4502A6Ab neg	**IFNω IFNα4**	het c.47C>T (T16M) het c.769C>T (R257X)	Calcitriol Magnesium supplement once a day Vitamin A, B6, B9, B12, C, D, zinc, iron, copper, and selenium supplement Calcium carbonate Omeprazole
11. (16)	F	28.7	13	CMC (0.1), CH (6), AD (8)	T1D (3.6), gastrointestinal dysfunction (3.9), temporal lobe epilepsy (4.7), hypercholesterolemia (5.6), constipation (5.8), ocular myasthenia (5.8), onychodystrophy (5.8), precocious puberty (6.6), HT (9.7), iron-deficiency anemia (20), secondary amenorrhea (21), alopecia (21), homo MTHFR mutation c.677C>T (22.6)	**TPHAb, AADC, TgAb, TPOAb, IAA, ICA, GADAb, IA2Ab, ACA, ovary Ab, 21OHAb pos** TRGAb, AGA, ANA, ANCA, SMA, EMA, AMA, ARA, LKMAb, LC1Ab, RAb, PCA, IFA, cardiolipin Ab, 17αOHAb, SCC Ab, cP4501A2Ab, cP4502D6Ab, cP4502A6Ab neg	**IFNω IFNα4 pos**	homo c.415C>T (R139X)	Insulin aspart Insulin degludec Hydrocortisone Fludrocortisone Calcitriol Calcium carbonate Clobazam Levetiracetam Lacosamide Carbamazepine Pyridostigmine bromide Lansoprazole Ezetimibe/Simvastatin Estradiol valerate/dienogest
12. (17)	M	34.2	5	CMC (6), AD (15)	Onychosis (*Candida albicans*) (6), low-grade chronic active autoimmune hepatitis (8), hypertransaminasemia, hypergammaglobulinemia, obesity, and short stature (due to long-term steroid treatment) (12), central diabetes insipidus (15)	**TPHAb, AADC, SMA, LKMAb, 21OHAb (cP450c21Ab), cP4501A2Ab, cP4502D6Ab pos** TgAb, TPOAb, IAA, GADAb, IA2Ab, TRGAb, ANA, AMA, ARA, RAb, mitochondrial Ab, other organ and non-organ specific Ab, 17αOHAb, SCCAb, HarmoninAb, cP4502A6Ab neg	**IFNω pos** IFNα4 neg	homo c.1314-1326del13/insGT (D439CfsX61)	Fludrocortisone Prednisone Omeprazole Desmopressin
13.	F	22	19.7	CH (3), CMC (4)	Autoimmune hepatitis (4), hypertransaminasemia, hypergammaglobulinemia	**TgAb, TPOAb, LKMAb pos** IAA, GADAb, IA2Ab, TRGAb, ZnT8Ab, ACA, ANA, ANCA, SMA, EMA, LC1Ab, ASCAb, RAb, SLA-IgG, Sp100Ab, gp21Ab neg	ND	het C322fsX372/C449Xhet 964-976del13	Azathioprine Prednisone Calcium lactate/gluconate Calcitriol
14.	M	11.1	9	CH (4)	Urticarial vasculitis (0.8), vitiligo (7), autoimmune hepatitis (8), autism	**TPOAb, ACA, ANA pos** ANCA, SMA, AMA, ARA, RAb, LKMAb, LC1Ab, SLA-IgG, PCA neg	ND	het c.47C>T (T16M) het c.967_979del13 (L323SfsX51)	Azathioprine PTH Calcium carbonate Calcitriol

In brackets, the age of onset of disease symptoms is shown. In bold, positive values.

Het, heterozygous; homo, homozygous; neg, negative; pos, positive; ASCA, anti-Saccharomyces cerevisiae Abs; HT, Hashimoto thyroiditis; GH, growth hormone; PRES, posterior reversible encephalopathy syndrome; U, unit; IU, international unit; HRT, hormone replacement treatment.

*Mutations and polymorphisms.

The mean actual age was 16.5 years (range, 6.5–34.2). The mean age at referral was 4.8 years (range, 1–19.7). Ages at the presentation of the three major components of APECED, clinical manifestations, molecular genetics, and Ab profile of patients are analytically reported in **Table 1**. In **Table 1**, we have also summarized the minor APECED manifestations that have been detected so far in the whole series, as well as the results of both genetic analyses and Ab screening. Patient Nos. 2, 9, and 10 included in **Table 1** are described for the first time in the present report, whereas the remaining cases have already been reported elsewhere (13–19). The patients were recruited from the University Department of Pediatrics (DPUO), the Endocrinology Unit, and the Hepatology, Gastroenterology and Nutrition Unit of Children’s Hospital Bambino Gesù (OPBG) IRCCS, in Rome (patient Nos. 1–4 and 10–14 in **Table 1**); from the Unit of Endocrinology and Rare Endocrine Diseases of Bari University (patient Nos. 9 and 10 in **Table 1**); from the Pediatric Endocrinology Units of the Department of Mother and Child; from the Department of Translational Medical Sciences of the University Federico II of Naples (patient Nos. 5–7 in **Table 1**); and from the Department of Human Pathology of Adulthood and Childhood of the University of Messina (patient No. 8 in **Table 1**).

### 2.2 Study design and methods

The clinical history of the 14 patients, from the first cardinal manifestation to the APECED diagnosis, was retrospectively reconstructed based on questionnaires addressed to the families by the authors, whereas their subsequent clinical evolution, from the time of diagnosis onward, was assessed based on clinical records of the patients at our centers. For the diagnosis of APECED, we used the criteria proposed by Husebye et al. (12). Informed consent was obtained from all those who took part in the present study in accordance with the Declaration of Helsinki. The investigation was approved by the local Institutional Review Board (IRB) of OPBG, which regulates using of human samples for experimental studies (Study Protocol No. 1385_OPBG_2017).

### 2.3 Autoantibody screening

Ab screening was performed in all cases at the time of diagnosis and periodically repeated during follow-up. The patients’ sera were assayed for insulin-dependent diabetes mellitus (type 1 diabetes (T1D))-related Abs, i.e., islet cell antibodies (ICAs) by immunofluorescence (IFL), anti-glutamic acid decarboxylase isoform 65 (GADAb) (First anti-GAD ELISA RSR, Cardiff, UK), anti-tyrosine phosphatase-related islet antigen 2 (IA2Ab) (First anti-IA2 ELISA RSR), anti-insulin (IAA) (Medzyme Corporation, Montreal, QC, Canada), and anti-zinc transporter 8 Abs (ZnT8Ab) (anti-ZnT8 RSR) by enzyme-linked immunosorbent assay (ELISA); and for thyroid-related Abs, i.e., anti-thyrotropin (TSH)-receptor (TRAb) immunoassay, Immulite TSI (Siemens Healthcare, Tarrytown, NY, USA), thyroglobulin (TgAb), and thyroperoxidase (TPOAb) *via* electrochemiluminescence immunoassay (ECLIA) (Siemens, Erlangen, Germany). Celiac-disease-related Abs were screened by chemiluminescence (CLIA, Quanta-Flash-Werfen, Monza, Milan, Italy), i.e., anti-transglutaminase (TRGAb) (CLIA, Quanta-Flash-Werfen, Monza, Milan, Italy) and by fluorimetric enzyme-linked immunoassay (FEIA), i.e., extractable nuclear antigen (ENA, SSA/RoAb) (ELiA, Thermo Fisher, Waltham, MA, USA) and by IFL endomysial Ab (EMA) (Werfen, Barcelona, Spain). Specific hepatic Abs, i.e., anti-liver–kidney microsomal (LKMAb) and anti-liver cytosol type 1 (LC1Ab), anti-ribosomal (RAb), and PCA were measured by indirect IFL (KSL-Werfen), while anti-soluble liver antigen IgG (SLA-IgG) (Euroimmun Italia s.r.l., Padua, Italy) via ELISA and IFA (ELiA, Thermo Fisher) were assayed *via* FEIA. Abs against autoantigen cytochrome P450 21-hydroxylase (21OHAb), hepatic autoantigens cytochrome P4501A2 (cP4501A2Ab and CYP1A2Ab) and P4502D6 (cP4502D6Ab), anti-villin (VillinAb), anti-75kDa (HarmoninAb and AIE-75Ab), and anti-interferonα4 (IFNα4Ab) were tested by luciferase immunoprecipitation system (LIPS, S. Raffaele Hospital, Milan, Italy) (22). Abs to autoantigen adrenal cortex (ACA) cells by IFL (Medical Systems, Milan, Italy), Abs to adrenal and gonadal autoantigens, cytochrome P450 side chain cleavage enzyme (SCCAb), adrenal autoantigen cP450c17α-hydroxylase (17αOHAb), hepatic aromatic l-amino acid decarboxylase (AADC), and TPHAb were tested by specific methods as previously reported (23–25) (S. Raffaele Hospital, Milan, Italy). IFNωAb was assayed by RIA in collaboration with FIRS Laboratories RSR Ltd (Cardiff, UK).

Non-organ specific Abs anti-nuclear (ANA), anti-neutrophil cytoplasmic (ANCA) (Elettrochimica s.r.l., Mainate, Varese), anti-double-stranded DNA (anti-dsDNA) (bioMérieux Italia S.p.A, Bagno a Ripoli, Florence, Italy), anti-reticulin (ARA), anti-mitochondrial (AMA), and anti-smooth muscle cell (SMA) were also tested by IFL (KSL-Werfen). Anti-cardiolipin Ab (CAb) was tested by FEIA (ELIA Thermo Fisher), and anti-glycoprotein 210 Ab (gp210Ab), which is indicative of primary biliary cirrhosis, was tested by Dot Blot assay (Alifax, Polverara, Padua, Italy).

### 2.4 Genetic study

All 14 exons and flanking exon–intron boundaries of the *AIRE* gene were sequenced in the DNA of recruited patients (26) according to already described protocols (Genetic Analyzer 3500 Applied Biosystems HITACHI System, Thermo Fisher Scientific, Rodano, Italy). Next-generation sequencing (NGS) was applied to the analysis of DNA samples of patient Nos. 1 and 2, and data were analyzed with the DRAGHEN Germline v3 algorithm (Illumina, San Diego, CA, USA).

## 3 Results

**Figure 1:**
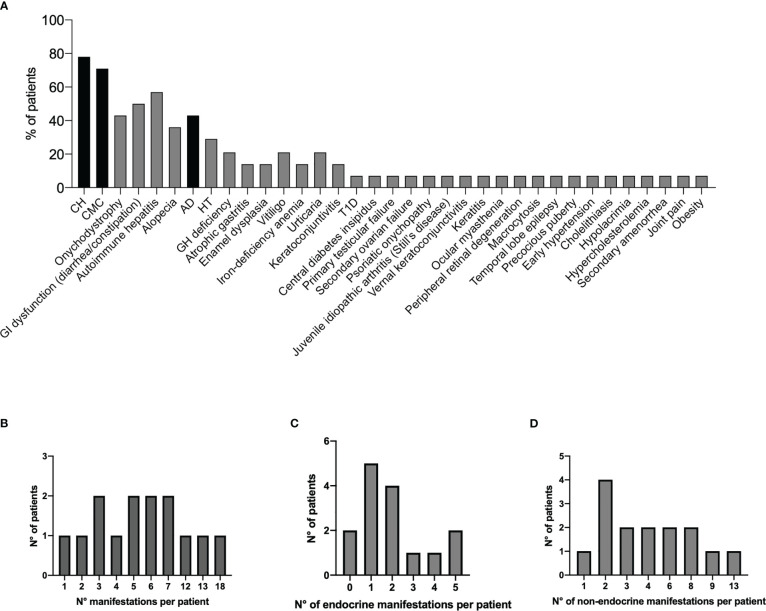
Prevalence of all disease manifestations in the 14 Italian APECED patients **(A)**. The black bars represent the classical diagnostic triad. Distribution of total clinical manifestations **(B)**, endocrinopathies **(C)**, and non-endocrine manifestations **(D)** per patient. APECED, autoimmune polyendocrinopathy-candidiasis-ectodermal dystrophy.

In the present series, all APECED patients presented APAH or autoimmune enteropathies (AIEs) (**Tables 1**, **2**, **Figure 1**, **Supplementary Figure 1**) (13–19). A description of unpublished case reports is reported in the **Supplementary Text**, **Supplementary Figure 1** and **Table 1**.

**Table 2:** Clinical manifestations, diagnostic criteria, and autoantibody tests for each disorder of the 14 APECED patients.

Disease	Main clinical manifestations	Diagnostic tests	Autoantibodies
Chronic mucocutaneous candidiasis (CMC)	Chronic candida infection of mucosae, nails, esophagus	Dermatological and ORL evaluation and culture	IFNAb, ILAb
Chronic hypoparathyroidism (CH)	Paraesthesia, tetany, muscle cramps, Trousseau or Chvostek’s signs	Calcium, PTH, ECG, EMG ischemic test	NALP-5Ab
Addison’s disease (AD)	Asthenia, hyperpigmentation, hypotension, weight loss, nausea	ACTH, cortisol, renin, ACTH test, ionemia	21OHAb, ACA
Primary ovarian failure	Amenorrhea less than 40 years, infertility	Estradiol, FSH, LH, anti-Müllerian hormone	StCA, 17OHAb, SCCAb, 21OHAb
Testicular failure	Infertility, erectile dysfunction	FSH, LH, testosterone	StCA, 17OHAb, SCCAb, 21OHAb
Autoimmune thyroid diseases	Clinical or subclinical hypothyroidism or hyperthyroidism	TSH, FT4, FT3, thyroid ultrasound	TPOAb, TgAb, TRAb
Type 1 diabetes	Polyuria, polydipsia, weight loss, coma	Glucose, C-peptide, HbA1c	ICA, GADAb, IA2Ab, ZnT8Ab
Pituitary deficiency	Signs/symptoms of hypothyroidism, hypogonadism, adrenal insufficiency, growth retardation	TSH, FT4, FSH, LH, estradiol, testosterone, ACTH, cortisol, HGH, IGF-1	Pituitary Ab, ECE-2Ab
Atrophic gastritis/pernicious anemia	Dyspepsia, microcytic or macrocytic anemia, asthenia	Blood count, iron, ferritin, vitamin B12, pepsinogen I, gastrin, gastric biopsy	PCA, PCA+IFA
Autoimmune intestinal disease	Chronic diarrhea or constipation	Low serotonin levels, gastric/duodenal biopsy	TPHAb, HDAb, AADC, VillinAb, HarmoninAb
Celiac disease	Diarrhea, abdominal bloating or pain, weight loss, intestinal malabsorption, slow growth (in children), anemia	Duodenal biopsy	TRGAb
Autoimmune hepatitis	Dyspepsia, fatigue, diarrhea, hepatomegaly	AST, ALT, GGT, ALP, bilirubin, liver ultrasound with biopsy	LC1Ab, SLA-IgG, cP4501A2Ab, cP4502D6Ab, cP4502A6Ab, ANA, SMA, ANCA, ActinAb, LKMAb
Vitiligo	Skin depigmentation	Dermatological clinical evaluation	SOXAb, MPCAb (present only in APECED)
Alopecia	Areata, total or universal, hair loss	Dermatological clinical evaluation	THAb (positive only in APECED)
Ectodermal dystrophy	Enamel hypoplasia, nail dystrophy, keratoconjunctivitis, tympanic calcifications, cataract, punctate nail defect, sublenticular cataract	Clinical evaluation	IFNAb
Hypokalemia with hypertension	Hypertension, muscle cramps	Na, K, Cl	IFNAb
Exocrine pancreas insufficiency	Diarrhea, malabsorption	Amylase, lipase	IFNAb

ORL, otorhinolaryngological (evaluation); ILAb, anti-interleukin Ab; NALP-5A, Nucleotide-Binding Oligomerization Domain, Leucine Rich Repeat and Pyrin Domain Containing 5 Ab; StCA, steroid cell Ab; ECE-2Ab, Endothelin Converting Enzyme 2 Ab; HDAb, histidine Ab; SOXAb, SRY-type related box Ab; MPCAb, melatonin-producing cell Ab; APECED, autoimmune polyendocrinopathy-candidiasis-ectodermal dystrophy; PTH, parathyroid hormone; ECG, electrocardiogram; EMG, electromyogram; ACTH, adrenocorticotropic hormone; FSH, follicle-stimulating hormone; LH, luteinizing hormone; TSH, thyroid-stimulating hormone; HbA1c, glycated hemoglobin; AST, aspartate aminotransferase; ALT, alanine aminotransferase; GGT, gamma-glutamyl transferase; ALP, alkaline phosphatase.

Nine patients (Nos. 1–5, 8, and 12–14) presented APAH (**Table 1**). The mean age of APAH diagnosis was 8.1 years (range, 1.5–13). In three patients (Nos. 1, 3, and 5), APAH occurrence was the first disease manifestation, while three patients (Nos. 2, 4, and 14) developed it following CH, two (Nos. 8 and 12) following CMC, and one (No. 13) developed APAH following CH and simultaneously with CMC. APAH-related specificities LKMAb, which target cytochrome P4502D6 (cP4502D6 and CYP2D6) autoantigen (27), tested positive in 3/9 patients (Nos. 4, 12, and 13) and TPH in 4/8 (Nos. 2, 5, 8, and 12); AADC was enriched in 6/7 (Nos. 1–3, 5, 8, and 12), cP450c21Ab in 4/6 (Nos. 1–4), and cP4501A2Ab in one patient (No. 12). Regarding other APAH-related Abs, ANA was present in 3/10 (Nos. 2, 3, and 14), SMA was present in 3/10 patients (Nos. 1, 3, and 12), ANCA and LC1Ab tested negative in all those tested (Nos. 1–5, 13, and 14 and Nos. 1–4, 13, and 14, respectively), and lastly, SLA-IgG was absent in all those tested (Nos. 1–4, 13, and 14) (**Table 1**). The most frequent *AIRE* mutation was c.967_979del13 (4/9 patients, Nos. 1, 3, 4, and 14) (10).

Seven patients (Nos. 5–11) were affected by signs of constipation, diarrhea, and/or malabsorption (**Table 1**). The mean age of intestinal dysfunction diagnosis was 6.1 years (range, 3.9–9). Two patients (Nos. 8 and 11) were also affected by iron-deficiency anemia and two (Nos. 5 and 10) by atrophic gastritis, and of them was affected with pernicious anemia (No. 10). The sera of five patients (Nos. 5, 7, 8, 10, and 11) tested positive for TPHAb and five (Nos. 5, 6, 8, 10, and 11) for AADC Abs known to be associated with AIE in APECED. AIE-75 and VillinAb tested negative in Nos. 5–10. cP450c21Ab tested positive in 3/6 patients (Nos. 6, 7, and 9) with gastrointestinal dysfunction. PCA was positive in 1/7 (No. 5), while IFA was present in 1/5 (No. 10) patients (**Table 1**). Six of six patients’ sera (Nos. 5–10) tested positive for IFNωAb and IFN-α4Ab (2). None of the patients presented celiac-related Abs. No specific *AIRE* genotype was prevalent.

Eleven of 12 (Nos. 1–8 and 10–12) of the whole series were positive for IFNω and 11/12 (Nos. 1–11) for IFNα4Ab.

Two patients (Nos. 5 and 8) with AIE also presented clinical laboratory parameters indicative of autoimmune hepatitis (*vide supra*).

## 4 Discussion

In this study, we have characterized the clinical presentation and autoantibody profile of APAH and gastrointestinal manifestations in a series of 14 Italian APECED patients. Of these, nine patients (Nos. 1, 3–8, 11, and 12) were previously reported by our group (13–19), while five patients were newly described in the present study (Nos. 2, 9, 10, 13, and 14) and were added to the published series of 158 APECED patients recruited from across Italy (14).

In the analysis conducted by Garelli et al. (14) on 568 patients from different ethnicities, APAH occurs with an overall prevalence of between 4% and 43% of patients. In particular, in the Italian APECED series, APAH was diagnosed in 21.5% of patients by the end of the follow-up (14). Autoimmune intestinal diseases were diagnosed between 5% and 54% of APECED patients and autoimmune gastritis/pernicious anemia in 4% to 45% of patients. In the Italian APECED series, 29.7% of patients developed autoimmune intestinal dysfunction; autoimmune gastritis was diagnosed in 25.9% of APECED patients, and pernicious anemia was diagnosed in 21/41 of patients.

The analysis of the present Italian APECED series showed a significant expansion of disease manifestations with 35 clinical entities in varying frequencies (from 7% to 78%, **Figure 1A**, **Table 1**) with a median of 5.5 manifestations per patient (mean 6.6; range, 1–18; **Figure 1B**). The observed spectrum and frequency of endocrine and non-endocrine manifestations (median of 1.5 endocrine manifestations per patient; mean 2; range, 0–5 (**Figure 1C**); median of 3.5 non-endocrine manifestations; mean 4.6; range, 1–13 (**Figure 1D**), respectively) were similar to those of published cohorts (rev in 12). Further, the putative association between eight manifestations and APECED/*AIRE* is highly debatable; these include IgA deficit, septic arthritis, periodic rash-associated fever, kidney cysts, headaches, polyps, posterior reversible encephalopathy syndrome (PRES), and abscesses.

Of note, organ-specific non-endocrine manifestations were enriched in the present series with autoimmune hepatitis and gastrointestinal dysfunction seen in 57% (814) and 50% (7/14) of patients, respectively (**Supplementary Figure 1**). Remarkably, in published cohorts, circulating autoantibodies were found in approximately 80% of non-APECED patients affected by AIH (28). Two clinically distinct subtypes of AIH can be distinguished based on Ab patterns: AIH-1 and AIH-2 (29). AIH-1 patients had the presence of circulating ANA and/or SMA. Antibodies directed against SLA/liver-pancreas antigen (LP) (30, 31) specifically occurred in AIH patients. Abs against neutrophilic antigens can be at high prevalence (32). A little overlap was found between AIH-1 and AIH-2 patients (28, 29). Patients affected by AIH-2 were characterized by LKM-1Ab. Often associated with AIH-2 was LC1 and less frequently Abs against UDP-glucuronosyltransferases (LKM3Ab). The prevalence and diagnostic value of autoantibodies associated with idiopathic AIH remain to be determined for patients with APAH. In APECED, molecular targets of different hepatic autoantigens have been identified: P4501A2 (33, 34), cytochrome P4502A6 (CYP2A6) (35/36), and AADC (35) (*vide supra*). Remarkably, AADCs were detected in 92% of Finnish patients with APAH, but they were also frequent in those with no other signs of APAH (35).

Overall autoantibodies against TPHAb and AADC were significantly associated in APECED patients of the present series (**Table 1**). The last AADC specificity was also significantly associated with autoimmune biopsy-proven hepatopathy. CYP1A2 and AADC are known hepatic autoantigens in APECED (34). CYP1A2Ab was detected in the serum of 4/8 patients with APAH and CYP2A6Ab in the serum of 3/8 patients. Overall, these data confirm the association with APAH discovered in the previous series (27). As regards CYP2A6 and CYP1A2, they are two of six cytochromes P450 that are highly expressed in human liver microsomes (36). CYP1A2 seems to be not expressed in extrahepatic tissues, while CYP2A6 is also expressed in human kidneys (37). CYP2D6Ab was detected in the serum of two patients with APAH (Nos. 3 and 12); in one case, it was associated with CYP1A2Ab. As regards CYP2D6, it is the major autoantigen of LKM-1Ab widely used as a diagnostic marker of AIH-2, which was detectable in 95%–100% of patients (28).

Clinical enteropathy was diagnosed in seven patients; AADC Abs tested positive in 5/7 patients, indicating gastrointestinal dysfunction in APECED. Consistent with published literature (38), the presence of TPHAb was significantly associated with APECED being present in the serum of 5/7 patients. In contrast, VillinAb and HarmoninAb tested negative, as they were reported to be detectable only in a few APECED patients, while these are specific markers of IPEX (39).

Among other cP450 disease-related specificities, adrenal and steroid cell antibodies are detectable in APECED patients and directed against cP450ssc and cP450c17 (17αOH) (40–42). In the present study, 5/12 patients had cP450sscAb and 2/12 had cP450c17Ab. In the literature, there is some disagreement on whether cP450c21 is an autoantigen in APECED. As regards this matter, Uibo et al. (1994) reported that this specificity is present in 15/50 APECED patients (42), while Winqvist (1995) (43, 44) and Clemente in the Sardinian cohort failed to detect it (33). In our series, we confirmed the presence of cP450c21Ab in 9/12 APECED patients.

Almost all patients with APECED usually develop high levels of neutralizing Ab anti-type 1 interferons, which are often detectable before the appearance of clinical symptoms of autoimmunity (40). Indeed, 11/14 patients of the present series (except for patient Nos. 9) showed circulating IFNωAb, and 11/14 patients showed circulating IFNα4Ab; in two patients, IFNAb was not investigated.

On a general ground, a genotype/phenotype correlation has been rarely observed in the APECED series (rev in 10). In the present small series of APECED patients, the most frequent *AIRE* mutation was c.967_979del13, which was detected in the DNA of 4/9 patients with APAH as previously reported (10). No specific *AIRE* genotype was prevalent in APECED patients with gastrointestinal manifestations: this does not allow to establish a clear genotype/phenotype correlation.

Although the application of Ferre-Lionakis expanded criteria (11, 12) would require validation in independent large cohorts of patients, the early enrichment of organ-specific non-endocrine manifestations, similar to the American cohort investigated, has allowed an expedited clinical diagnosis in 11/14 patients (Nos. 1–5, 8–12, and 14) of the present series. On a general ground based on Ferre-Lionakis criteria, the presence of APAH or gastrointestinal manifestations in association with one symptom of the classical triad could lead to a clinical diagnosis that should be further confirmed by *AIRE* gene sequencing and detection of IFNωAb (11). This emphasizes the importance in the clinical practice of the definition of APAH and gastrointestinal dysfunction for an earlier diagnosis of APECED even in European cohorts. An earlier diagnosis would also allow the prevention of episodes of life-threatening hypocalcemic seizures and adrenal crisis, which are the main manifestations of undiagnosed APECED.

## Data availability statement

The datasets presented in this article are not readily available because of ethical consent for patients. All reported mutations of the *AIRE* gene are already described in the literature. Requests to access the datasets should be directed to corresponding authors and clinicians Marco Cappa, Maria Felicia Faienza, Giuseppe Maggiore, Mariacarolina Salerno.

## Ethics statement

The studies involving human participants were reviewed and approved by Bambino Gesù Children’s Hospital, IRCCS, Rome, Italy. Written informed consent to participate in this study was provided by the participants’ legal guardian/next of kin.

## Author contributions

GP, OP, AC, and EC analyzed data and conducted genetic screening. MF, MC, AP, DC, MV, GM, and MS provided patients with clinical data and contributed to writing the manuscript. VL conducted hepatopathy and enteropathy-related Ab determinations. CB contributed to writing the manuscript and provided critical revision. AF designed the study; analyzed, interpreted, and discussed the clinical and laboratory data; and wrote the manuscript. All authors contributed to the article and approved the submitted version.
