# Supplementary material for: Analysis of a series of Italian APECED patients with autoimmune hepatitis and gastro-enteropathies
Source: Front Immunol. 2023 Jun 30;14:1172369. doi: 10.3389/fimmu.2023.1172369 (PMC10348686; doi:10.3389/fimmu.2023.1172369)
Supplement: Supplementary file 1 [file DataSheet_1.pdf]

Supplementary Material

Analysis of a Series of Italian APECED Patients with Autoimmune Hepatitis and Gastro-Enteropathies

Giorgia Paldino[1], Maria Felicia Faienza[2], Marco Cappa[3], Andrea Pietrobattista[4], Donatella Capalbo[5], Mariella Valenzise[6], Vito Lampasona[7], Annamaria Cudini[1], Elena Carbone[1], Olivia Pagliarosi[1], Giuseppe Maggiore[4], Mariacarolina Salerno[8], Corrado Betterle[9], Alessandra Fierabracci[1]*

*** Correspondence:** alessandra.fierabracci@opbg.net

1 Supplementary Text

Patient No 2 (female, actual age 14.1 years) was referred at the age of 7 years for symptoms related to CH and Hashimoto's thyroiditis (HT), leading to hormonal substitutive treatment for these endocrine conditions. At the age of 10 years, she was diagnosed with Vernal keratoconjunctivitis and with vitiligo at 11 years old. At 12 years of age, she developed partial IgA deficit. At the age of 13 years, she was diagnosed with mucocutaneous candidiasis and chronic autoimmune hepatitis for the presence of hypertransaminasemia and hypergammaglobulinemia. Liver histology evidenced a dense lymphomononuclear and eosinophil inflammatory infiltrate with plasma cellular infiltration of the liver limiting plate. In addition to ANA, SSAAb, 17αOHAb, TPHAb, AADC, ACA and cP450c21Ab were indicative of the risk of autoimmune intestinal disease. AD and premature menopause in APECED tested positive. T1D-related specificities IAA, GADAb, IA2Ab and celiac disease-related TRGAb tested negative. APECED diagnosis was confirmed by the presence of IFNωAb and IFNα4Ab and by the presence of the compound *AIRE* heterozygous mutations c.769C>T (p.Arg257Ter or R257X) in exon 6 and c.1616C>T (p.Pro539Leu or P539L) in exon 14.

Patient No 9 (female, actual age 14.2 years) presented nail dystrophy and alopecia at referral at the age of 1 year, followed by oral CMC and abscesses at 4 years. She developed CH at the age of 7 years, followed by joint pain at the age of 8 years and chronic mucous diarrhea at 9. Among Abs specificities TgAb, TPOAb, IAA, ANA, cP450c21Ab and cP4502A6Ab were found positive without signs of specific diseases but being at risk of hypothyroidism, T1D and AD. However, GADAb, IA2Ab, ovaryAb, 17OHAb, SCCAb, cP4501A2Ab and cP4502D6Ab were negative. Homozygous *AIRE* c.64_69delGTGGAC (p.Val22_Asp23del or V22_D23del) mutation in exon 1 and IFNα4Ab positivity confirmed APECED diagnosis.

Patient No 10 (female, age at referral 1 year and actual age 9.5 years) developed CMC and Still's disease (with the presence of HLA DR11 DQ7 predisposing alleles) at the age of 1 year; mucous diarrhea and psoriatic onychopathy at the age of 6 years; macrocytosis at the age of 7 years; CH at the age of 8 years and atrophic gastritis at the age of 9 years. EGDS revealed bulbitis and nodular hyperplasia of the terminal ileum, esophagitis and atrophic gastritis. GADAb, ANA, TPHAb, AADC, IFA all tested positive. Homozygous methylenetetrahydrofolate reductase (MTHFR) c.677C>T mutation was revealed, predisposing to preclinical homocystinemia. APECED was diagnosed by *AIRE*

gene screening detecting the compound heterozygosity c.47C>T (p.Thr16Met or T16M) in exon 1 and R257X in exon 6 and by testing positive for IFNα4Ab and for IFNωAb.

Patient No 13 (female, age at presentation 19.7 years and actual age of 22 years) presented hypertonic seizures at the age of 22 months and CH was diagnosed at the age of 3 years. She developed diffuse CMC at the age of 4 years that was treated with systemic anti-fungal drugs. At the age of 4 years, due to the presence of hypertransaminasemia and hypergammaglobulinemia the patient was diagnosed autoimmune hepatitis and tested positive for LKMAb. Liver biopsy revealed preserved architecture with diffuse portal space inflammation and eosinophils and plasma cell infiltration. Confluent necrosis with central portal bridges at the lobular level, high interface activity with neoductulogenesis and ductular metaplasia of hepatocytes were observed. The patient was responsive to immunosuppressive therapy.

Patient No 14 (male, age at presentation 9 and actual age 11) is affected by autism and developed urticarial vasculitis at the age of 10 months, chronic hypoparathyroidism at the age of 4 years and vitiligo at the age of 7 years. At the age of 8 years, autoimmune hepatitis was diagnosed confirmed by liver biopsy showing low-grade fibrosis and tested positive for ANA and negative for LKMAb. The patient was responsive to immunosuppressive therapy. APECED diagnosis was confirmed by *AIRE* gene screening with the compound heterozygosity c.47C>T/ c.967_979del13.

2 Supplementary Figures and Tables

2.1 Supplementary Figures

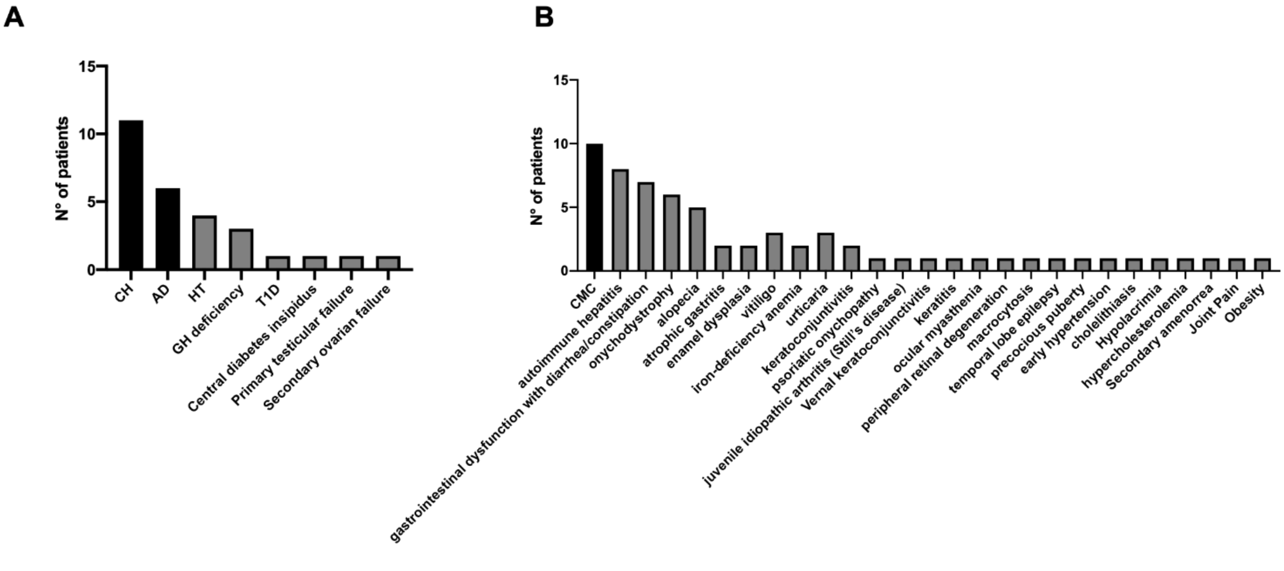

Supplementary Figure 1. Distribution of all clinical endocrine (A) and non-endocrine (B) manifestations among the 14 patients. The black bars represent the classical diagnostic triad.

2.2 Supplementary Tables

Supplementary Table 1. Methods used for autoantibodies measurements

IFL	ELISA	CLIA	FEIA	Recombinant immunoassay	DOT Blot	LIPS
ICA	GADAb	TgAb	ENA	TRAb	Gp210Ab	21OHAb
LKMAb	IA2	TPOAb	SSA/RoAb	IFNω		cP4501A2Ab
LC1Ab	IAA	TRGAb		SSCAb		cP4502D6Ab
RAb	ZnT8Ab			cP450c17α		cP4502A6Ab
PCA	SLA-IgG			AADC		VillinAb
ACA				TRHAb		HarmoninAb
ANA						
ANCA						
dsDNA						
ARA						
AMA						
SMA						
EMA						
